# Growth Hormone Receptor Gene is Essential for Chicken Mitochondrial Function In Vivo and In Vitro

**DOI:** 10.3390/ijms20071608

**Published:** 2019-03-31

**Authors:** Bowen Hu, Shuang Hu, Minmin Yang, Zhiying Liao, Dexiang Zhang, Qingbin Luo, Xiquan Zhang, Hongmei Li

**Affiliations:** 1 Department of Animal Genetics, Breeding and Reproduction, College of Animal Science, South China Agricultural University, Guangzhou 510642, China; wind7318@stu.scau.edu.cn (B.H.); hushuang@stu.scau.edu.cn (S.H.); Minminyang@stu.scau.edu.cn (M.Y.); 20183139032@stu.scau.edu.cn (Z.L.); zhangdexiang0001@sina.com (D.Z.); qbluo@scau.edu.cn (Q.L.); 2 Guangdong Provincial Key Lab of AgroAnimal Genomics and Molecular Breeding and Key Lab of Chicken Genetics, Breeding and Reproduction, Ministry of Agriculture, Guangzhou 510642, China

**Keywords:** growth hormone receptor, mitochondrial function, sex-linked dwarf chicken, skeletal muscle, DF-1 cells

## Abstract

The growth hormone receptor (*GHR*) gene is correlated with many phenotypic and physiological alternations in chicken, such as shorter shanks, lower body weight and muscle mass loss. However, the role of the *GHR* gene in mitochondrial function remains unknown in poultry. In this study, we assessed the function of mitochondria in sex-linked dwarf (SLD) chicken skeletal muscle and interfered with the expression of *GHR* in DF-1 cells to investigate the role of the *GHR* gene in chicken mitochondrial function both in vivo and in vitro. We found that the expression of key regulators of mitochondrial biogenesis and mitochondrial DNA (mtDNA)-encoded oxidative phosphorylation (OXPHOS) genes were downregulated and accompanied by reduced enzymatic activity of OXPHOS complexes in SLD chicken skeletal muscle and *GHR* knockdown cells. Then, we assessed mitochondrial function by measuring mitochondrial membrane potential (ΔΨm), mitochondrial swelling, reactive oxygen species (ROS) production, malondialdehyde (MDA) levels, ATP levels and the mitochondrial respiratory control ratio (RCR), and found that mitochondrial function was impaired in SLD chicken skeletal muscle and *GHR* knockdown cells. In addition, we also studied the morphology and structure of mitochondria in *GHR* knockdown cells by transmission electron microscopy (TEM) and MitoTracker staining. We found that knockdown of *GHR* could reduce mitochondrial number and alter mitochondrial structure in DF-1 cells. Above all, we demonstrated for the first time that the *GHR* gene is essential for chicken mitochondrial function in vivo and in vitro.

## 1. Introduction

Mitochondria are dynamic organelles with a crucial role in cellular energy homeostasis and metabolism, with the generation of adenosine triphosphate (ATP) through the respiratory chain (RC) being one of their main functions [1]. The RC is composed of four complexes (I–IV) embedded in the inner mitochondrial membrane which are uniquely controlled by mitochondrial DNA (mtDNA) and the nuclear genome [2]. Similar to mammalian mtDNA, chicken mtDNA determines 37 gene products, of which 13 are oxidative phosphorylation (OXPHOS) subunits (complex I, III, IV and V) [3]. In addition, mitochondrial biogenesis is essential for its function, which is mainly regulated by nuclear genes through the PGC1α–NRF1–TFAM signaling pathway [4]. Peroxisome proliferator-activated receptor γ co-activator 1α (PGC1α), the master regulator of mitochondrial biogenesis, can activate the expression of nuclear respiratory factor-1 (NRF1) and mitochondrial transcription factor A (TFAM) to regulate mtDNA replication and transcription in mammals [5].

Growth hormone (GH) and growth hormone receptor (GHR) come together in the GH–GHR–IGF1 signaling pathway to influence mitochondrial function in mammals. Growth hormone can regulate mitochondrial function through the Box 1 region of the *GHR* gene in CHO cells [6], and several alterations in OXPHOS complexes have been observed in GH-deficient Ames mice [7]. Knockout of *GHR* (GHRKO) in mice could also alter the expression of key regulators of mitochondrial biogenesis [8,9]. Furthermore, previous studies have reported that insulin-like growth factor 1 (IGF1) signaling can regulate mitochondrial biogenesis markers in the steroidogenic cells of prepubertal testis [10], and is essential for mitochondrial biogenesis in cancer cells [11]. These findings suggest that the *GHR* gene plays a pivotal role in mammalian mitochondrial function both in vivo and in vitro.

Sex-linked dwarf (SLD) chickens, which are characterized by diverse mutations in *GHR* [12], are feasible models for understanding the role of *GHR* in poultry [13]. Previous studies have reported that these mutations in *GHR* can result in many phenotypic and physiological alterations in chicken, such as shorter shanks, lower body weight and muscle mass loss [14,15,16]. However, the role of *GHR* in mitochondrial function in poultry remains unknown.

Based on the abovementioned findings, we assessed the function of mitochondria in SLD chicken skeletal muscle and interfered with the expression of *GHR* in DF-1 cells to investigate the role of *GHR* in chicken mitochondrial function both in vivo and in vitro. Here, we demonstrate for the first time that *GHR* is essential for chicken mitochondrial function both in vivo and in vitro.

## 2. Results

### 2.1. Low Expression of Key Regulators of Mitochondrial Biogenesis and mtDNA-Encoded OXPHOS Genes in SLD Chicken Skeletal Muscle

**Figure 1:**
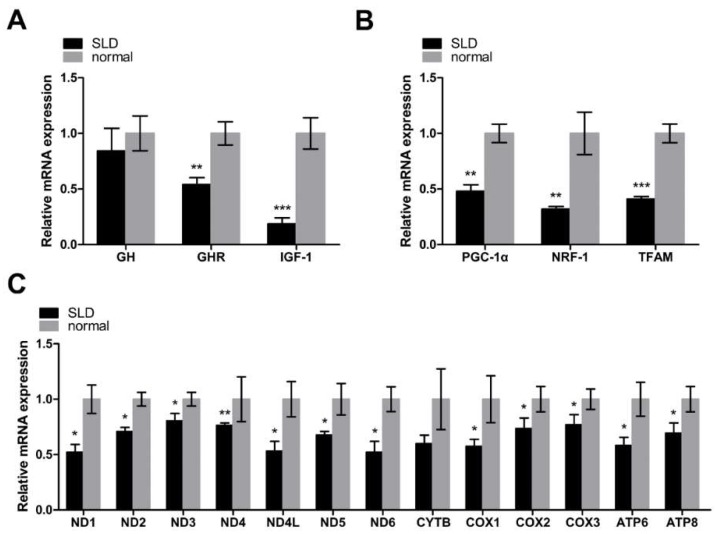
Low expression of key regulators of mitochondrial biogenesis and mtDNA-encoded oxidative phosphorylation (OXPHOS) genes in sex-linked dwarf (SLD) chicken skeletal muscle. (**A**) The relative mRNA expression of genes involved in the GH–GHR–IGF1 signaling pathway was measured by qRT-PCR in SLD chicken skeletal muscle as compared with normal chicken skeletal muscle. (**B**) The relative mRNA expression involved in the PGC1α–NRF1–TFAM signaling pathway was measured by qRT-PCR in SLD chicken skeletal muscle as compared with normal chicken skeletal muscle. (**C**) The relative mRNA expression of mtDNA-encoded OXPHOS genes was measured by qRT-PCR in SLD chicken skeletal muscle as compared with normal chicken skeletal muscle. Data are expressed as means ± SEM, * *p* < 0.05; ** *p* < 0.01; *** *p* < 0.001.

In this study, we first assessed the relative mRNA expression of genes involved in the GH–GHR–IGF1 signaling pathway by qRT-PCR. The relative mRNA levels of the *GH* gene were not significantly different, however, for *GHR* and *IGF1*, were significantly downregulated in SLD chicken skeletal muscle compared with normal chicken skeletal muscle, indicating the low level of GH binding activity in SLD chicken skeletal muscle (Figure 1A). In order to investigate the role of *GHR* in mitochondrial biogenesis in vivo, we assessed the relative mRNA expression of the genes involved in the PGC1α–NRF1–TFAM signaling pathway using qRT-PCR. We found the relative mRNA expression of *PGC1α*, *NRF1*, and *TFAM* to all be significantly downregulated in SLD chicken skeletal muscle compared with normal chicken skeletal muscle (Figure 1B), indicating an inhibition of mitochondrial biogenesis in SLD chicken skeletal muscle. Since TFAM can regulate the transcription of mtDNA, we then determined the relative mRNA expression of mtDNA-encoded OXPHOS genes. We found that the expression of mtDNA-encoded OXPHOS genes was also downregulated in SLD chicken skeletal muscle compared with normal chicken skeletal muscle, indicating that mtDNA transcription was inhibited in SLD chicken skeletal muscle; however, we did not observe a significant difference in *CYTB* expression (Figure 1C). Taken together, we found low expression of key regulators of mitochondrial biogenesis and mtDNA-encoded OXPHOS genes in SLD chicken skeletal muscle.

### 2.2. Reduced Enzymatic Activity of OXPHOS Complexes in SLD Chicken Skeletal Muscle

**Figure 2:**
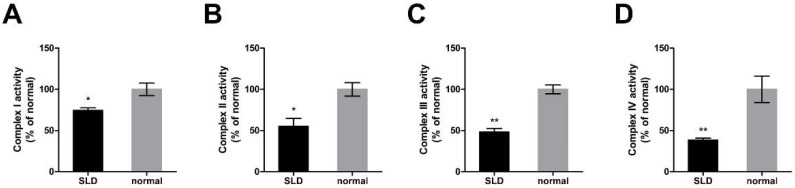
Reduced enzymatic activity of OXPHOS complexes in SLD chicken skeletal muscle. (**A**) Enzymatic activity of complex I was measured by the change in absorbance of NADH in SLD and normal chicken skeletal muscle. (**B**) Enzymatic activity of complex II was measured by the change in absorbance of DCIP in SLD and normal chicken skeletal muscle. (**C**) Enzymatic activity of complex III was measured by the change in absorbance of reduced cytochrome c in SLD and normal chicken skeletal muscle. (**D**) Enzymatic activity of complex IV was measured by the change in absorbance of reduced cytochrome c in SLD and normal chicken skeletal muscle. Data are expressed as means ± SEM, * *p* < 0.05; ** *p* < 0.01; *** *p* < 0.001.

To investigate the role of *GHR* on the enzymatic activity of OXPHOS proteins in vivo, we assessed the enzymatic activity of OXPHOS complexes in SLD chicken skeletal muscle. We found that the enzymatic activities of OXPHOS complex I, II, III and IV were significantly reduced by 26%, 45%, 53% and 72% in SLD chicken skeletal muscle compared with normal chicken skeletal muscle, respectively (Figure 2A–D), indicating the reduced enzymatic activity of OXPHOS complexes in SLD chicken skeletal muscle.

### 2.3. Impaired Mitochondrial Function in SLD Chicken Skeletal Muscle

**Figure 3:**
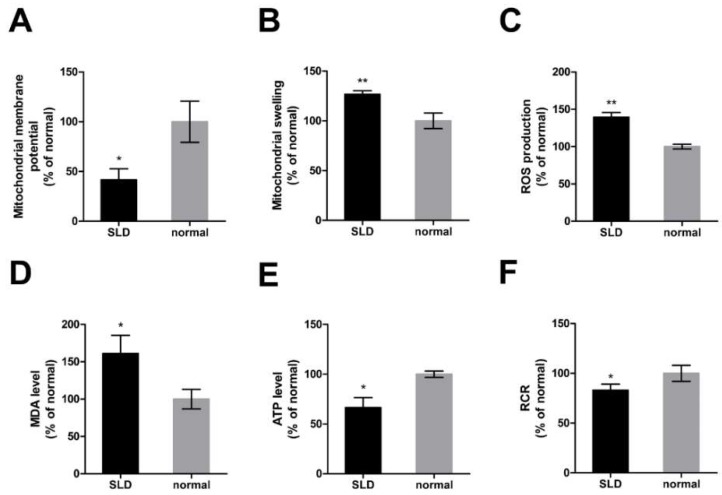
Impaired mitochondrial function in SLD chicken skeletal muscle. (**A**) Mitochondrial ΔΨm was measured by the fluorescence of JC-1 in SLD and normal chicken skeletal muscle. Red fluorescence represents aggregation of JC-1, green fluorescence represents monomeric JC-1, and ΔΨm was represented as the ratio of aggregated and monomeric JC-1. (**B**) Mitochondrial swelling was measured by the absorbance at 540 nm of mitochondria isolated from SLD and normal chicken skeletal muscle. (**C**) Reactive oxygen species (ROS) production was measured by the fluorescence of dichlorofluorescein (DCF) in SLD and normal chicken skeletal muscle. The level of (**D**) malondialdehyde (MDA) and (**E**) ATP were measured in SLD and normal chicken skeletal muscle. (**F**) Mitochondrial respiratory control ratio (RCR) was calculated as the ratio of state III to state IV respiration rate in SLD and normal chicken skeletal muscle. Data are expressed as means ± SEM, *p < 0.05; ** *p* < 0.01; *** *p* < 0.001.

To further investigate the role of *GHR* in chicken mitochondrial function in vivo, we assessed mitochondrial function in SLD and normal chicken skeletal muscle by measuring mitochondrial membrane potential (ΔΨm), mitochondrial swelling, reactive oxygen species (ROS) production, malondialdehyde (MDA) levels, ATP levels, and the mitochondrial respiratory control ratio (RCR). ΔΨm was significantly reduced (Figure 3A) and accompanied by a significant increase of mitochondrial swelling (Figure 3B) in SLD chicken skeletal muscle compared with normal chicken skeletal muscle. Reactive oxygen species production was significantly increased (Figure 3C) and accompanied by a significant increase of MDA level (Figure 3D) and a significant reduction of ATP level (Figure 3E) in SLD chicken skeletal muscle compared with normal chicken skeletal muscle. Consistently, RCR was significantly reduced in SLD chicken skeletal muscle compared with normal chicken (Figure 3F). Results showed that mitochondrial function in SLD chicken skeletal muscle was impaired.

### 2.4. Knockdown of GHR Downregulated the Expression of Key Regulators of Mitochondrial Biogenesis and mtDNA-Encoded OXPHOS Genes in DF-1 Cells

**Figure 4:**
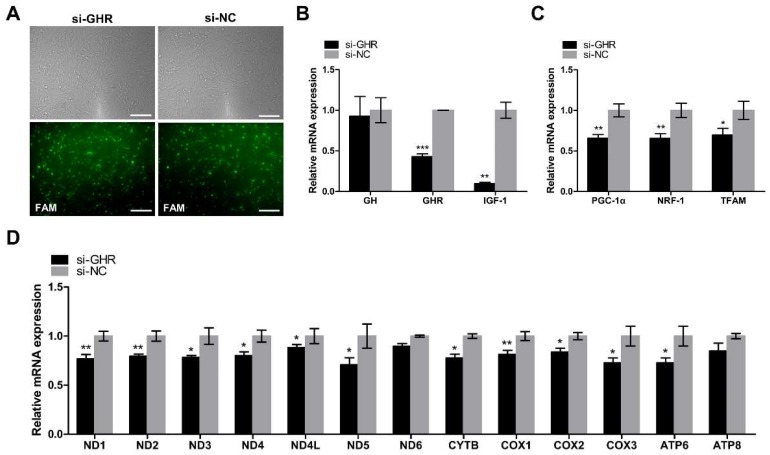
Knockdown of *GHR* reduced the GH binding activity and downregulated the expression of key regulators of mitochondrial biogenesis and mtDNA-encoded genes in DF-1 cells. (**A**) Transfection efficiency was measured by the fluorescence intensity of FAM siRNA and qRT-PCR at 48 h after transfection with si-GHR and si-NC fragments in DF-1 cells. Bar, 100 μm. The relative mRNA expression of genes involved in the GH–GHR–IGF1 signaling pathway (**B**) and PGC1α–NRF1–TFAM signaling pathway (**C**) were measured by qRT-PCR at 48 h after transfection with si-GHR and si-NC fragments in DF-1 cells. (**D**) mtDNA transcription was measured by qRT-PCR at 48 h after transfection with si-GHR and si-NC fragments in DF-1 cells. Data are expressed as means ± SEM, * *p* < 0.05; ** *p* < 0.01; *** *p* < 0.001.

In order to determine the role of *GHR* in chicken mitochondrial function in vitro, we interfered with the expression of *GHR* in DF-1 cells. Transfection efficiency was measured by the fluorescence intensity of FAM siRNA and qRT-PCR. The expression of the *GHR* gene was significantly downregulated in *GHR* knockdown cells, indicating that we had successfully interfered with the expression of the *GHR* gene in DF-1 cells (Figure 4A,B). Then, we detected the relative mRNA expression of the genes involved in the GH–GHR–IGF1 signaling pathway. We found that the expression of *GH* was not significantly different in *GHR* knockdown cells, however, the expression of *IGF1* was significantly downregulated in *GHR* knockdown cells, indicating the low level of GH binding activity in *GHR* knockdown cells (Figure 4B). In order to investigate the role of *GHR* in mitochondrial biogenesis in vitro, we then detected the expression of key regulators of mitochondrial biogenesis and mtDNA-encoded OXPHOS genes in DF-1 cells. *PGC1α*, *NRF1*, *TFAM*, and mtDNA-encoded OXPHOS genes were all downregulated in *GHR* knockdown cells, but we did not observe a significant difference in *ND6* and *ATP8* expression (Figure 4C,D). Taken together, we demonstrated that knockdown of *GHR* downregulated the expression of key regulators of mitochondrial biogenesis and mtDNA-encoded OXPHOS genes in DF-1 cells.

### 2.5. Knockdown of GHR Reduced the Enzymatic Activity of OXPHOS Complexes in DF-1 Cells

**Figure 5:**
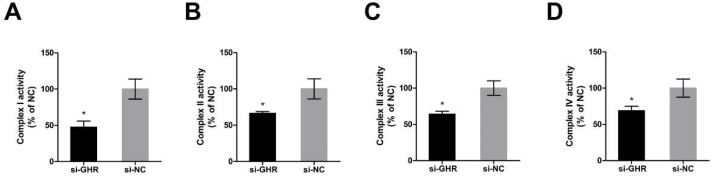
Knockdown of *GHR* reduced the enzymatic activity of OXPHOS complexes in DF-1 cells. (**A**) Enzymatic activity of complex I was measured by the change in absorbance of NADH at 48 h after transfection with si-GHR and si-NC fragments in DF-1 cells. (**B**) Enzymatic activity of complex II was measured by the change in absorbance of DCIP at 48 h after transfection with si-GHR and si-NC fragments in DF-1 cells. (**C**) Enzymatic activity of complex III was measured by the change in absorbance of reduced cytochrome c at 48 h after transfection with si-GHR and si-NC fragments in DF-1 cells. (**D**) Enzymatic activity of complex IV was measured by the change in absorbance of reduced cytochrome c at 48 h after transfection with si-GHR and si-NC fragments in DF-1 cells. Data are expressed as means ± SEM, * *p* < 0.05; ** *p* < 0.01; *** *p* < 0.001.

In order to investigate whether the knockdown of *GHR* could alter the enzymatic activities of OXPHOS proteins in DF-1 cells, we assessed the enzymatic activity of OXPHOS complexes at 48 h after transfection with si-GHR and si-NC fragments in DF-1 cells. The enzymatic activities of OXPHOS complex I, II, III and IV were significantly reduced by 53%, 34%, 36% and 25% in *GHR* knockdown cells, respectively (Figure 5A–D), indicating that the knockdown of *GHR* could reduce the enzymatic activity of OXPHOS complexes in DF-1 cells.

### 2.6. Knockdown of GHR Impaired Mitochondrial Function in DF-1 Cells

**Figure 6:**
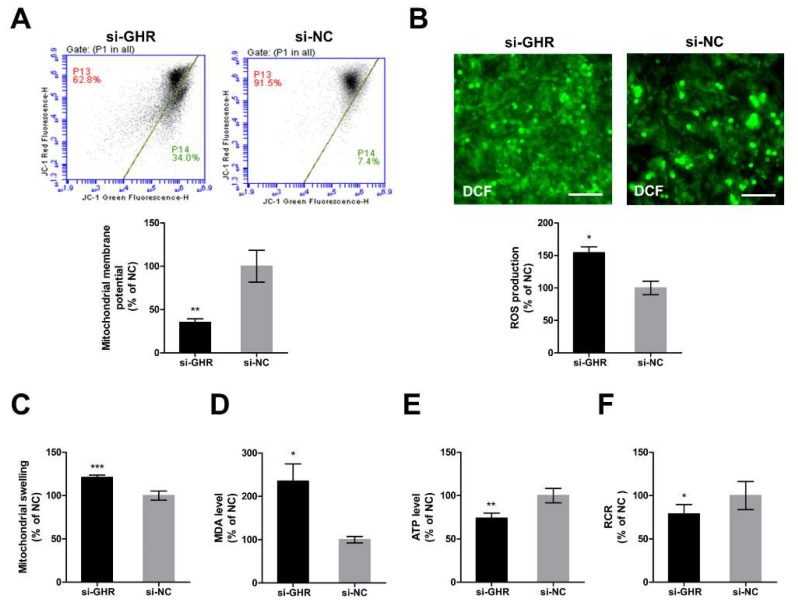
Knockdown of *GHR* caused mitochondrial dysfunction in DF-1 cells. (**A**) Mitochondrial ΔΨm was measured by the fluorescence of JC-1 at 48 h after transfection with si-GHR and si-NC fragments in DF-1 cells. Red fluorescence represents aggregation of JC-1, green fluorescence represents monomeric JC-1, ΔΨm was represented as the ratio of aggregated and monomeric JC-1. (**B**) Reactive oxygen species production was measured by the fluorescence of DCF at 48 h after transfection with si-GHR and si-NC fragments in DF-1 cells. Bar, 100 μm. (**C**) Mitochondrial swelling was measured by the absorbance at 540 nm after transfection with si-GHR and si-NC fragments in DF-1 cells. The level of (**D**) MDA and (**E**) ATP were measured at 48 h after transfection with si-GHR and si-NC fragments in DF-1 cells. (**F**) Respiratory control ratio was calculated as the ratio of state III to state IV respiration rate at 48 h after transfection with si-GHR and si-NC fragments in DF-1 cells. Data are expressed as means ± SEM, * *p* < 0.05; ** *p* < 0.01; *** *p* < 0.001.

In order to further investigate whether the knockdown of *GHR* could cause mitochondrial dysfunction in DF-1 cells, we assessed mitochondrial function in *GHR* knockdown cells. ΔΨm was significantly reduced (Figure 6A) and accompanied by a significant increase of mitochondrial swelling (Figure 6C) in *GHR* knockdown cells. Reactive oxygen species production was significantly increased (Figure 6B) and accompanied by a significant increase of MDA level (Figure 6D) and a significant reduction of ATP level (Figure 6E) in *GHR* knockdown cells. Consistently, RCR was significantly reduced in *GHR* knockdown cells (Figure 6F). As a result, we demonstrated that the knockdown of *GHR* impaired mitochondrial function in DF-1 cells.

### 2.7. Knockdown of GHR Altered Mitochondrial Structure and Reduced Mitochondrial Number in DF-1 Cells

**Figure 7:**
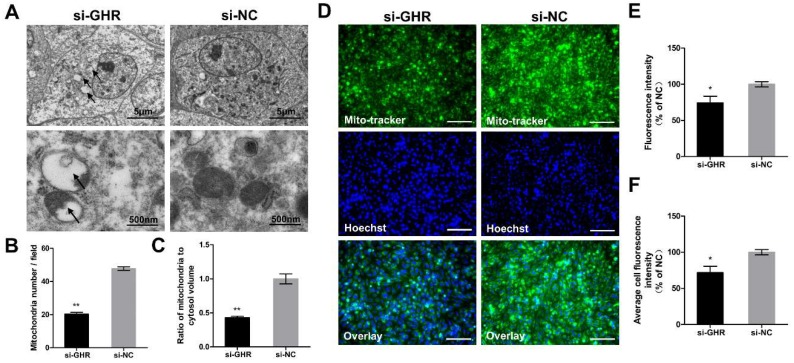
Knockdown of *GHR* altered mitochondrial structure and reduced mitochondrial number in DF-1 cells. (**A**) Mitochondrial ultrastructure was imaged by TEM at 48 h after transfection with si-GHR and si-NC fragments in DF-1 cells, and abnormal mitochondria are indicated by black arrows. (**B**) The number of mitochondria and (**C**) the ratio of mitochondria to cytosol volume as determined from TEM images were assessed in DF-1 cells (*n* = 5). (**D**) MitoTracker staining of DF-1 cells was measured at 48 h after transfection with si-GHR and si-NC fragments in DF-1 cells. Bar, 100 μm. (**E**) Fluorescence intensity of MitoTracker staining and (**F**) average cell fluorescence intensity of MitoTracker staining were assessed in DF-1 cells. Data are expressed as means ± SEM, * *p* < 0.05; ** *p* < 0.01; *** *p* < 0.001.

Considering that the morphology and structure of mitochondria lays the foundation for mitochondrial function, we assessed them by TEM at 48 h after transfection with si-GHR and si-NC fragments in DF-1 cells. We found some alterations in structure of mitochondria in *GHR* knockdown cells (see arrows), including increased mitochondrial vacuolation, absence of dense matrix granules and disappearance of mitochondria cristae (Figure 7A). We also found that knockdown of *GHR* could significantly reduce mitochondrial number and the ratio of mitochondria to cytosol volume in DF-1 cells by TEM (Figure 7B,C). In order to verify the consequences of TEM, we then labeled the mitochondria by MitoTracker staining, in which fluorescence intensity represented the mitochondrial mass [17]. The mitochondrial mass was significantly reduced in *GHR* knockdown cells, which was consistent with the result of TEM (Figure 7D–F). In summary, we demonstrated that knockdown of *GHR* could reduce mitochondrial number and alter mitochondrial structure in DF-1 cells.

## 3. Discussion

Growth hormone can bind to GHR to regulate IGF-1 production through the JAK–STAT pathway [18]. The mutation in *GHR* in SLD chickens interferes with the binding between GH and GHR, and further disrupts the GH–GHR–IGF1 signaling pathway, accompanied by low levels of IGF-1 [19]. A previous study has reported that compared with aging rats that had been administrated with exogenous IGF-1, untreated aging rats showed significant mitochondrial dysfunction [20], indicating that the level of IGF1 is essential for mammalian mitochondrial function. In this study, we found that the relative mRNA expression of *IGF1* was significantly downregulated in both SLD chickens and *GHR* knockdown cells, indicating the low level of GH binding activity and IGF-1 production. These results further prompted us to investigate mitochondrial function in SLD chicken skeletal muscle and in *GHR* knockdown cells.

Mitochondrial biogenesis, a dynamically regulated process, is mainly regulated by nuclear genes through the PGC1α–NRF1–TFAM signaling pathway [4]. The activity of PGC1α is related to its expression level [21], and the low expression of *PGC1α* is associated with mitochondrial dysfunction in human skeletal muscle [22]. PGC1α can regulate expression of TFAM, and the overexpression of *TFAM* can stimulate mitochondrial biogenesis in mice fibroblast cells [23]. The role of TFAM has been highlighted by regulating the transcription of mtDNA-encoded genes in human skeletal muscle [24]. Our results are consistent with these findings in mammals because we also found that the relative mRNA expression of *PGC1α*, *TFAM* and mtDNA-encoded OXPHOS genes were all downregulated in SLD chicken skeletal muscle and *GHR* knockdown cells. Furthermore, there is evidence of low expression of *PGC1α* and reduced enzymatic activity of OXPHOS complexes in the skeletal muscle of GH-deficient Ames mice [7], and downregulated relative mRNA expression of *NRF1* and *TFAM* in the skeletal muscle of GHRKO mice [8]. These findings are also consistent with our results, in which we observed the low expression of genes involved in the PGC1α–NRF1–TFAM signaling pathway and reduced enzymatic activity of OXPHOS complexes in SLD chicken skeletal muscle. However, our results show that the enzymatic activity of complexes I, II, III and IV was reduced by 26%, 45%, 53% and 72% in SLD chicken skeletal muscle, respectively, while in *GHR* knockdown cells the enzymatic activity was reduced by 53%, 34%, 36% and 25%, respectively, that means an exactly opposite trend. This is probably due to the mutation in *GHR* in vivo that is different from the interference with *GHR* in vitro. The mutation in *GHR* completely alters the function of the *GHR* in vivo, which might lead to a larger impact on the activity of complex IV. As the interference with *GHR* cannot completely inhibit the function of the *GHR* in vitro, which might primarily affect the activity of complex I, we argue that the regulatory mechanism of the enzymatic activities of OXPHOS complexes might be different between SLD chicken skeletal muscle and *GHR* knockdown cells.

Impaired mitochondrial biogenesis and inhibited mtDNA transcription will further lead to mitochondrial dysfunction in mammals [25]. ΔΨm is essential for mitochondrial function; loss of ΔΨm indicates mitochondrial dysfunction [26] and is normally accompanied by increased mitochondrial swelling [27]. Reduced ΔΨm may lead to uncoupling of OXPHOS, and the impairment of OXPHOS complex activities may increase ROS production accompanied by elevated MDA levels and reduced ATP levels [28]. Meanwhile, RCR is a sensitive indicator of mitochondrial respiratory function [29]. In cases of hepatic mitochondrial dysfunction in a murine model of peanut allergy, decreased RCR can be observed with a simultaneous increase in ROS production [30]. Here, we found that reduced ΔΨm, increased mitochondrial swelling, excessive ROS production, elevated MDA levels, reduced ATP levels and decreased RCR were observed in SLD chicken skeletal muscle and *GHR* knockdown cells. Our results are in line with previous studies in mammals showing that the function of mitochondria is compromised in GHRKO osteocytes [31] and inhibition of IGF1 signaling leads to mitochondrial dysfunction in cancer cells [11]. Therefore, we argue that the low expression of *IGF1* might cause mitochondrial dysfunction by regulating mitochondrial biogenesis through the PGC1α–NRF1–TFAM signaling pathway in poultry.

In addition, the morphology and structure of mitochondria are essential for mitochondrial function and cell homeostasis [32]. The results of this study showed that knockdown of *GHR* and the accompanying low expression of *IGF1* could reduce the number of mitochondria and alter the structure of mitochondria, as observed in DF-1 cells as increased mitochondrial vacuolation, an absence of dense matrix granules and the disappearance of mitochondria cristae. Our results are in agreement with a previous study showing that a change in IGF1 signaling could alter the morphology and structure of mitochondria in mice Leydig cells [10].

In conclusion, we demonstrated for the first time that the function of mitochondria was impaired in SLD chicken skeletal muscle and *GHR* knockdown cells, and knockdown of *GHR* can reduce mitochondrial number and alter the structure of mitochondria in DF-1 cells. We conclude that the *GHR* gene is essential for chicken mitochondrial function both in vivo and in vitro.

## 4. Materials and Methods

### 4.1. Ethics Statement

All animal experiments in this study were performed according to the protocols approved by the South China Agriculture University Institutional Animal Care and Use Committee (approval number: SCAU#0015). All animal procedures followed the regulations and guidelines established by this committee and minimized the suffering of animals.

### 4.2. Animals

In this study, we used 7-week-old female SLD chickens in strain N301 as the experimental group, and this strain is characterized by a T354C mutation in exon 5 of *GHR* as previously described [14]. Meanwhile, we used normal 7-week-old female chickens in strain N202 as a control group, which have a wild-type *GHR* gene. Here, we used gastrocnemius muscle in SLD chickens to assess the role of *GHR* in chicken mitochondrial function in vivo. All chickens were obtained from the WenShi Group Co., Ltd. (Guangdong, China).

### 4.3. Cell Culture and RNA Interference

In this study, we interfered with the expression of *GHR* using a chicken embryo fibroblast (DF-1) cell line to assess the role of *GHR* in chicken mitochondrial function in vitro. The DF-1 cells were cultured in high-glucose Dulbecco’s modified Eagle’s medium (Gibco, NY, USA) with 10% fetal bovine serum (Hyclone, UT, USA) and 0.2% penicillin/streptomycin (Invitrogen, CA, USA). The DF-1 cells were plated on a culture plate and incubated overnight prior to the transfection experiment. The siRNAs used for the knockdown of *GHR* were synthesized by Guangzhou RiboBio (Guangzhou, China). In our preliminary experiments, we designed four siRNA to interfere with *GHR* and used the si-GHR with the highest interference efficiency. The sequence of si-GHR was 5′-CCUCGAUUUGGAUACCAUA-3′. si-GHR and si-NC were transfected in DF-1 cells to a final concentration of 20 nM using Lipofectamine 3000 reagent (Invitrogen, CA, USA) according to the manufacturer’s protocol, and cells were analyzed at 48 h after transfection. The *GHR* inhibition efficiencies were detected by the fluorescence intensity of FAM siRNA and qRT-PCR.

### 4.4. Quantitative Real-Time PCR

Total RNA was extracted from gastrocnemius muscle or cells with RNAiso reagent (Takara, Japan) according to the manufacturer’s protocol. The RNA integrity and concentration were determined using 1.5% agarose gel electrophoresis and a Nanodrop 2000c spectrophotometer (Thermo, USA), respectively. cDNA was synthesized using PrimeScript RT Reagent Kit (Takara, Japan) for qRT-PCR. The MonAmp™ ChemoHS qPCR Mix (Monad Co., LTD Guangzhou, China) was used for quantitative real-time PCR (qRT-PCR) in a Bio-Rad CFX96 Real-Time Detection instrument (Bio-Rad, Hercules, CA, USA) according to the manufacturer’s protocol. Relative gene expression was measured by qRT-PCR twice for each reaction and the nuclear gene *β-actin* was used as a control. The primers used in qRT-PCR are shown in Table S1.

### 4.5. Transmission Electron Microscopy

TEM was used to determine the morphology and structure of mitochondria. Cells were fixed in 2.5% glutaraldehyde for 4 h at 4 °C and then cultured as previously described [16]. A transmission electron microscope (Hitachi HT7700, Tokyo, Japan) was used to examine and photograph mitochondria, and five randomly selected areas were photographed at 2500× magnification and counted as previously reported [33].

### 4.6. MitoTracker Green Staining and Hoechst 33342 Staining

MitoTracker Green staining and Hoechst 33342 staining was used to label the mitochondria and nuclei in DF-1 cells, respectively. Cells were washed twice with PBS and incubated with MitoTracker Green (Beyotime, Shanghai, China) for 30 min at 48 h after transfection. Cells were then suspended in PBS and 10 μL of Hoechst 33342 dye was added (Beyotime, Shanghai, China). After being washed in PBS twice, a fluorescence microscope (Nikon TE2000-U, Tokyo, Japan) was used to capture five randomly selected fields and analyzed with NIS-Elements software.

### 4.7. Mitochondria Isolation and Mitochondrial Protein Concentration Measurement

The mitochondria of gastrocnemius muscle and DF-1 cells were isolated using mitochondrial extraction kits (C3606, C3601; Beyotime, Shanghai, China) according to the manufacturer’s protocol, as previously described [34]. Mitochondrial protein concentration was measured by BCA protein assay to normalize the protein content as previously described [35].

### 4.8. Measurement of Enzymatic Activity of Mitochondrial OXPHOS Complexes

Gastrocnemius muscle was dissected and immediately frozen in liquid nitrogen, and then stored at −80 °C [36]. We used commercial assay kits (BC0515, BC3235, BC3245, BC0945; Solarbio, Beijing, China) to measure the enzyme activity of mitochondrial OXPHOS complexes of gastrocnemius muscle and DF-1 cells according to the manufacturer’s protocol. Complex I enzyme activity was determined by the change in absorbance of NADH as measured at 340 nm. Complex II enzyme activity was determined by the change in absorbance of DCIP as measured at 600 nm. The enzyme activity of complex III and complex IV was determined by the change in absorbance of reduced cytochrome c as measured at 550 nm. Absorbance was determined using a Fluorescence/Multi-Detection Microplate Reader (BioTek, Winooski, VT, USA) according to the manufacturer’s protocol. Data were normalized to the control group and expressed as a percentage of control levels. 

### 4.9. Measurement of Adenosine Triphosphate Level

The ATP level was measured using an ATP assay kit (S0026; Beyotime, Shanghai, China) according to the manufacturer’s protocol. A Fluorescence/Multi-Detection Microplate Reader (BioTek, USA) was used to determine the ATP level in gastrocnemius muscle and cells as previously described [37]. Data were normalized to the control group and expressed as a percentage of control levels.

### 4.10. Measurement of Malondialdehyde Level

The MDA level was measured using an MDA assay kit (S0131; Beyotime, Shanghai, China) according to the manufacturer’s protocol. The supernatants of gastrocnemius muscle mitochondria and cell lysis were incubated with MDA reagent for 40 min at 95 °C. Absorbance was determined using a Fluorescence/Multi-Detection Microplate Reader (BioTek, USA). Data were normalized to the control group and expressed as a percentage of control levels.

### 4.11. Measurement of Mitochondrial Membrane Potential

Mitochondrial membrane potential was measured using a JC-1 kit (C2005; Beyotime, Shanghai, China) according to the manufacturer’s protocol. Gastrocnemius muscle mitochondria were fixed with JC-1. The fluorescence was determined using a Fluorescence/Multi-Detection Microplate Reader (BioTek, USA) and DF-1 cells were incubated with JC-1 for 20 min at 37 °C. After washing in PBS twice, the fluorescence was determined using a flow cytometer (BD Biosciences, San Jose, CA, USA), and 10 µM rotenone was used as standard inhibitor of ΔΨm. The ΔΨm of mitochondria were represented as the ratio of aggregated and monomeric JC-1, and data were normalized to the control group and expressed as a percentage of control levels.

### 4.12. Measurement of Mitochondrial Swelling

Mitochondrial swelling was measured by the absorbance at 540 nm, as previously described [38]. A decrease in absorbance indicates an increase in mitochondrial swelling. Mitochondria were freshly isolated from gastrocnemius muscle and DF-1 cells, and absorbance was determined using a Fluorescence/Multi-Detection Microplate Reader (BioTek, USA). Data were normalized to the control group and expressed as a percentage of control levels.

### 4.13. Measurement of Reactive Oxygen Species Production

Reactive oxygen species production of gastrocnemius muscle mitochondria was determined using the rate of NBT reduction at 595 nm, as previously described [39]. Absorbance was determined using a Fluorescence/Multi-Detection Microplate Reader (BioTek, USA). Reactive oxygen species production in the mitochondria of DF-1 cells was measured using a ROS Assay Kit (S0033; Beyotime, Shanghai, China) according to the manufacturer’s protocol. Cells were incubated with 10 mM DCFH-DA probes at 37 °C for 20 min and washed twice with PBS. Dichlorofluorescein (DCF) fluorescence was determined using a Fluorescence/Multi-Detection Microplate Reader (BioTek, USA), and the images of cells were taken by a fluorescence microscope (Nikon TE2000-U, Japan). Data were normalized to the control group and expressed as a percentage of control levels.

### 4.14. Measurement of Mitochondrial Respiratory Control Ratio

Mitochondrial respiratory control ratio was measured using an RCR kit (GMS10097; GenMed Scientifics Inc., MA, USA) according to the manufacturer’s protocol, as previously described [29]. Oxygen consumption of mitochondria protein was measured using a Clarke-type oxygen electrode (Hansatech Oxytherm, Norfolk, UK). The RCR was represented as the ratio of state III to state IV respiration rate. Data were normalized to the control group and expressed as a percentage of control levels.

### 4.15. Statistical Analysis

All experiments were performed at least three times. The data were presented as means ± standard error of the mean (SEM), the statistical analyses were performed using Student’s *t*-test, and the significance was represented by *p*-values. *p* < 0.05 was considered to be statistically significant, * *p* < 0.05, ** *p* < 0.01, *** *p* < 0.001.

## Supplementary Materials

Supplementary materials can be found at https://www.mdpi.com/1422-0067/20/7/1608/s1.
