# Supplementary material for: Growth Hormone Receptor Gene is Essential for Chicken Mitochondrial Function In Vivo and In Vitro
Source: Int J Mol Sci. 2019 Mar 31;20(7):1608. doi: 10.3390/ijms20071608 (PMC6480491; doi:10.3390/ijms20071608)
Supplement: Supplementary file 1 [file ijms-20-01608-s001.pdf]

## Supplemental Information

**Table S1.** Primers used in qRT-PCR.

| Gene | Primer sequence (5′ to 3 ′) | Size | Annealing temp. (°C) |
|---|---|---|---|
| F-ND1 | ACCCAAGAGCCCATCTACCT | 154 | 56 |
| R-ND1 | GTCCGGCGGCATATTCTACA | | |
| F-ND2 | CCGAGCGATTGAAGCCACTA | 103 | 56 |
| R-ND2 | TCATTGTCCGGTGGATCAGG | | |
| F-ND3 | CACCACTATCATCGCCCTCC | 72 | 56 |
| R-ND3 | TCTGCTCATTCTAAGCCGCC | | |
| F-ND4 | AGCGCAGGCATTTACCTTCT | 144 | 56 |
| R-ND4 | TGTTCAGGAGGCAGGTAGGT | | |
| F-ND4L | ATGTCTCCCCTACACTTCAGC | 82 | 56 |
| R-ND4L | AGATGAGGTGTGTTCGGTGG | | |
| F-ND5 | CTAATCCTAGCCGCCACAGG | 79 | 56 |
| R-ND5 | GGGTTGGGCCTTCTATTGCT | | |
| F-ND6 | ACCCAAACTCACTAACCACCC | 88 | 56 |
| R-ND6 | TGTGGCGTCTAATCCTTCGC | | |
| F-CYTB | CAGCAGACACATCCCTAGCC | 104 | 56 |
| R-CYTB | GAAGAATGAGGCGCCGTTTG | | |
| F-COX1 | ACTACTTACCGACCGCAACC | 132 | 56 |
| R-COX1 | CCGAAACCTGGGAGGATGAG | | |
| F-COX2 | TCGGGGTAAAAACAGACGCA | 70 | 56 |
| R-COX2 | ACTCCTGGTCGAGTGGTGAT | | |
| F-COX3 | CCCAAGCCCATGACCAATCT | 168 | 56 |
| R-COX3 | TGGAAGGTGCTTTCTCGGAC | | |
| F-ATP6 | TACAGCCACAATCGCCCTAC | 123 | 56 |
| R-ATP6 | AGGACGAAGACGTAGGCTTG | | |
| F-ATP8 | AACCCAAACCCATGATTCTCCA | 139 | 56 |

| | | | |
|---|---|---|---|
| R-ATP8 | AGGTTCAGGGGGTGGGTTTA | | |
| F-PGC1$\alpha$ | TCCTTTCCTCAACGCAGGTC | 153 | 56 |
| R-PGC1$\alpha$ | TCTTGCACGTGAGGGAGAAC | | |
| F-NRF1 | ACGAGGACTCACCTTCCTCA | 163 | 56 |
| R-NRF1 | TGTGGTCGCTTCCGTTTCTT | | |
| F-TFAM | GACCTCGAAGTGGCTTCAAC | 144 | 56 |
| R-TFAM | GAGCAAGCTGAAGGTATGGCT | | |
| F-GH | TGCAATACCTAAGCAAGGTGTTCA | 101 | 62 |
| R-GH | CAGGGCTTGGATCCCTTCTT | | |
| F-GHR | GCAAGTGCAGGTCACCTGAG | 153 | 56 |
| R-GHR | CCGGACATTCTTTCCAGTCT | | |
| F-IGF1 | TTGGCCTGTGTTTGCTTACC | 265 | 56 |
| R-IGF1 | ATTCCCTTGTGGTGTAAGCGT | | |
| F-βactin | GATATTGCTGCGCTCGTTG | 178 | 56 |
| R-βactin | TTCAGGGTCAGGATACCTCTTT | | |
